# Commentary: What should referral pathways have to improve healthcare experiences of women with female genital mutilation in Australia?

**DOI:** 10.1186/s12978-021-01274-w

**Published:** 2021-11-07

**Authors:** Carolyne Njue, Edward K. Ameyaw, Bright O. Ahinkorah, Abdul-Aziz Seidu, Samuel Kimani

**Affiliations:** 1 School of Public Health, University of Technology Sydney, PO Box 123, Broadway, NSW 2007 Australia; 2 College of Public Health, Medical and Veterinary Sciences, James Cook University, Townsville, QLD Australia; 3 University of Nairobi and Africa Coordinating Centre for Abandonment of FGM/C (ACCAF), PO Box 19676-00202, Nairobi, Kenya

**Keywords:** Female genital mutilation, Female circumcision, Referral pathways, FGM-related care and management, Australia

## Abstract

**Background:**

We examined the evidence derived from healthcare professionals’ interfacing with women with female genital mutilation (FGM) to comprehend the referral pathways available to these women in Australia.

**Main body:**

Clinicians encountered FGM-related complications that included ruptured bladder and total urinary incontinence. Midwives and paediatricians indicated a lack of referral pathways for FGM, but used their discretion to refer such cases to social work departments, obstetric/gynaecological units, child protection service providers, psychological counsellors and surgeons. The continuum of care for women with FGM is characterised by inadequate and lack of clear referral pathways. This underscores the need to develop and strengthen referral pathways in response to physical, birthing and psychological complications of women with FGM to improve their care experiences in Australia.

**Short conclusion:**

Capacity building initiatives on FGM-prevention and care for trainees and practising health providers and community involvement in high burden areas/populations should be implemented to promote uptake and utilization of the referral services. Provision of infrastructural support, including clinical management tools, job aids, posters, referral algorithms and electronic patient records with "drop-down menus" for referral sites for health complications of FGM to reinforce the providers’ efforts are critical.

## Background

Female genital mutilation (FGM) is prevalent in more than 90 countries worldwide; mainly in communities across 28 African countries (across the West, East and Northeast), the Middle East, Latin America and Asia [1–3] and diaspora communities [4]. The rationales for practising FGM vary across communities but are often deeply rooted in culture, traditions and beliefs [3]. According to the World Health Organisation, the practice of FGM involves procedures that entail the partial or total removal of the external female genitalia or other injuries for non-therapeutic reasons [5]. This ranges from the total or partial removal of: clitoris and/or the prepuce (clitoridectomy), clitoris and the labia minora with or without excision of the labia majora (excision), narrowing of the vaginal orifice with creation of a covering seal by cutting and appositioning the labia minora and/or the labia majora with or without excision of the clitoris (infibulation). It also comprises other harmful procedures to the female genitalia for non-medical reasons such as: pricking, piercing, scraping and cauterization [1, 5]. Globally, the practice of FGM violates well-established human rights principles, norms, and standards, including the violation of girls and women’s right to physical integrity and freedom from violence [6, 7]. The practice of FGM prevents girls and women from achieving the highest attainable health standards and is a form of discrimination [8, 9]. The prevalence of FGM among Australian women is not known [10]. However, it is estimated that 53,000 females born elsewhere but living in Australia have undergone FGM (i.e. rate of 4.3 per 1000 girls and women in Australia) [11]. Therefore, it is critical to prioritise groups at risk and those who have suffered FGM induced harm individually or collectively to respond to their health, psychological and human rights needs.

Globally, UNICEF estimates that more than 200 million girls and women have undergone some form of FGM, with more than 3.6 million girls at risk of being cut every year [12]. About 44 million FGM survivors are under 15 years old, with most having been cut younger than age five. The practice of FGM has vast negative health impacts. In the short term, girls and women with FGM may experience bleeding, genito-urinary infections, pain, shock and other significant psychological trauma immediately after the procedure [3, 13, 14]. Many experience long-term consequences, including gynaecological, birthing, sexual and psychological complications [3, 15]. These complications lead to women and girls seeking healthcare services, but evidence shows that health systems face challenges responding to these impacts due to limited infrastructural or skills capacity necessitating referrals [16].

As more women with FGM settle in Australia, they are more likely to have adverse obstetric outcomes than those without the procedure. As a result, they may require well-coordinated healthcare, including practical and responsive referral pathways to health facilities and clinicians with requisite skills to optimise the health of women affected by FGM during pregnancy and childbirth. An understanding of these referral pathways and the strategies developed to manage referrals in Australia are crucial. Consequently, the objective of this study is to synthesise empirical evidence on FGM referral pathways in Australia.

### Obtaining literature for the commentary

Initially, five electronic databases were searched for original articles: PubMed, Medline, CINAHL, Embase and Cochrane Library. A strategy for specific search terms was developed: "FGM/C" OR "FGC" OR "FC" OR "Female Genital Mutilation" OR "Female Genital Cutting" OR "Female Circumcision", OR "Referral pathways" OR "Referral options" OR "Referral processes" and "Australia". Only studies published in English were included, with no limit on the timeline. Database searches were supplemented with hand searching of the reference lists of the included studies. All identified studies were subjected to citation searches using all citations produced by Google Scholar. We further searched institutional websites and databases of organisations involved in FGM interventions or maternity-pregnancy and birth care to identify reports and any possible 'grey literature'.

### Referral pathways: what is the current situation?

Studies show that women and girls who migrate from FGM prevalent countries and live in high-income settings may be subjected to the practice while visiting their home countries [17, 18]. High-income countries of migration have notably prioritised prevention, protection and care interventions to address social injustice and protect those at risk of FGM. Additionally, these settings have implemented community-based activities that favour health persuasion, promote outreach services, involve community champions’ and include professional training and capacity-strengthening programs for healthcare professionals [19]. Some training on FGM may reflect the Royal Australian and New Zealand College of Psychiatrists (RANZCP) guidelines, including best practice guidelines for referral and communication for general practitioners and psychiatrists [20].

Importantly, new tools and approaches have been developed, with countries instituting clinical recommendations, specialised clinics, and training resources for health care workers to better care for affected women and girls [21, 22]. For example, the Green Top Guidelines on FGM by the Royal College of Obstetricians and Gynaecologists in Australia advises that all clinicians should be aware of FGM complications. The guidelines also recommend that midwives, obstetricians and gynaecologists serving FGM cases/populations should receive compulsory training to manage FGM [23]. It is critical that countries have well-coordinated FGM-related care, including practical and responsive referral systems, as well as clinicians who are equipped with the requisite skills to optimise the health of women affected by FGM across the lifespan, including during pregnancy and childbirth.

Although well-coordinated FGM-related care is important, information about  practical access to specialised care and clear referral pathways for migrant women with FGM in Australia is limited. Empirical evidence on FGM in Australia has mainly focused on research into health professionals' knowledge, predominantly among midwives working with women with FGM [24, 25] and paediatricians [26, 27]. These studies revealed mixed findings, highlighting that some midwives have limited knowledge about FGM [25] while others suggest that few of these professionals could identify the health issues presented by women living with FGM in their care [24]. One study that examined records of women with FGM in Australia identified some health complications, namely ruptured bladder and total urinary incontinence [28]. This highlights the need for health professionals to have skills to address these complications, be conversant and/or access the referral system to efficiently respond to the clients' needs.

Moreover, evidence shows that a greater proportion of healthcare professionals did not know the referral pathways for women with FGM [25]. In one study, nearly half of the surveyed midwives (46%; n = 76/165) did not know the referral path for a pregnant woman with FGM [25]. The healthcare professionals who had some level of knowledge about referrals for women with FGM stated that women with FGM were mainly referred to obstetric and gynaecological units [27]. Based on the healthcare providers' discretion, referral paths were to the social work department, child protection services and allied health professionals including counsellors and clinicians such as an obstetricians, specialists, general practitioners (GPs), surgeons or other clinics and hospitals [25, 27, 28]. Therefore, clear protocols for a referral system are urgently needed to resonate with specific needs of clients with FGM-related complications.

The aforementioned findings into healthcare providers’ knowledge of and encounters with FGM demonstrate the need to have a clear and well-defined referral pathway for FGM and associated complications, as well as build capacity for healthcare workers within the Australian health community. It is urgent and critical that midwives be facilitated through capacity building using evidence-based information on well-defined referral pathways for managing pregnant women with FGM [24–26]. Most women in Australia with FGM are migrants, usually of African descent [29, 30], and some may have accessed the country as survivors through asylum seeking [31]. As refugees and asylum seekers, many may already deal with psychological challenges, including FGM-related events and other issues affecting health literacy [32, 33]. A package to manage this should focus on referrals and comprise specific FGM-related interventions, prevention, psychosocial support and information about available services. Several state-specific tools including guidelines [34, 35] and national recommendations [36] for managing FGM need effective implementation to translate them into practical solutions for those in need.

An overview of health systems worldwide indicates that Australia’s healthcare system is one of the most comprehensive globally [37] and includes an elaborate in-built referral system. This provides an array of services ranging from general and preventative health to complex conditions that may require treatment from specialists. The health system has a public and private component, with funding mainly from the government, private health insurers and patients’ out-of-pocket expenses [37]. The federal government’s healthcare funding—Medicare—has been Australia’s universal healthcare scheme since 1984 and has three main aspects, being medicines, medical services and public hospitals [37]. Challenges to the Australian health system include increasing prevalence of chronic disease, cost of medical research and innovation, and the need to better apply health data [37]. Although the Australian healthcare system is exemplar, it is encountering challenges such as FGM, linked to global movements associated with asylum-seeking, migration, and forced population shifts [10, 32, 38]. This is compounded by limited referral systems for the clients with FGM-related complications [39].

The management of FGM-related complications should involve a clear and efficient referral system. A consistent and well-coordinated referral pathway for FGM-related services in Australia should focus on prevention, protection and care services for women/girls at risk of FGM. These interventions are offered through critical sectors of health, psychosocial support (such as spiritual care and counselling), public education and effective legal/justice services. While the health sector is executing a response to FGM, intra-system referrals are critical considering how diverse the level of complexity of care, specialities and professionals is along the continuum. For instance, whilst a client may require gynaecological services, another may need surgical and psychological care, while someone else may need all three combined services—interventions that would require different specialities, thus the need for referral. Moreover, the health system may identify women/girls who may require protection/access to justice, therefore invoking inter-system referrals through the justice/legal system to assist the client. For referral systems to be efficient, effective and seamless, community engagement and capacity building of health care providers is required. Additionally, a database with detailed information of all possible institutions and professionals to refer to - including names, addresses, telephone numbers and specialisation details is critical. This database should be shared with all health facilities to easily trigger referral mechanisms to avoid delays or barriers as well as to enable clients to call for information and to book appointments for FGM-related services. Finally, an effective monitoring and evaluation mechanism should be anchored in the referral protocol to help clarify the realities and trends within and across institutions by assessing referral system utility, efficiency, and prospects for improvement.

## Conclusions

There are various opportunities to enhance and improve on FGM safeguarding and early intervention services for girls and women with FGM. Health professionals would be more proficient with FGM-related referrals if a streamlined pathway is defined and promoted among those caring for women with FGM. A well-articulated referral pathway can enhance health professionals’ competencies in easily identifying and managing post-FGM complications. Extensive consultations and teamwork may be required to develop a tailored and responsive referral pathway. This may include a collaborative approach on FGM interventions between the Ministry of Health, other state government offices and local health promotion agencies. Furthermore, FGM-affected communities should be involved in developing an effective and receptive referral and reporting system for Australia to promote uptake, utility and value for money. Improved resources and an effective dissemination plan are vital to ensure the referral pathway's implementation and adherence. By doing so, service providers who previously had no or limited knowledge about FGM may improve their understanding and skills in how they identify, manage, counsel and refer women with FGM complications when they are encountered.

Additionally, a capacity-building plan should incorporate FGM-related care into pre-service and in-service training programs for general practice to augment health care providers' knowledge [26] to ensure accurate information and the ability to address ethical and legal aspects of FGM including cultural sensitivity in discussions with families of those with FGM. Engaging all cadres and levels of practitioners will ensure they are better positioned to offer streamlined, comprehensive and appropriate woman-centred maternity care and referrals. This will ensure holistic care, with well-defined efficient referral pathways that integrate intra-sector referral and inter-sector care services. This will ultimately promote prevention, protection and care services for women with FGM to achieve the best possible outcome.

## Data Availability

Not applicable.

## Abbreviations

FGM: Female genital mutilation
UNICEF: United Nations International Children's Emergency Fund
RANZCP: Royal Australian and New Zealand College of Psychiatrists
